# An Asymptomatic Sigmoid Colonic Fistula Arising from a Large Aneurysm of the Internal Iliac Artery Was Discovered during a Medical Examination

**DOI:** 10.3390/medicina60071052

**Published:** 2024-06-26

**Authors:** Myung Jo Kim, Kwon Cheol Yoo, Dae Hoon Kim

**Affiliations:** 1 Department of Surgery, College of Medicine, Chungbuk National University, Cheongju 28644, Republic of Korea; 2 Department of Surgery, Chungbuk National University Hospital, Cheongju 28644, Republic of Korea

**Keywords:** internal iliac artery aneurysm, rectal fistula, medical examination, case report

## Abstract

The rupture of an internal iliac artery aneurysm in the colon is a rare but potentially fatal complication. We report a rectal fistula of an asymptomatic internal iliac artery aneurysm that was discovered incidentally during a medical examination. A 77-year-old man presented at a local hospital for a general medical examination. Although the blood reports revealed severe anemia, the patient did not complain of any associated symptoms including dizziness and hematochezia. Moreover, there was no palpable mass in the patient’s abdomen, and there was no evidence of hematochezia, as the patient had been using a bidet. Interestingly, computed tomography (CT) revealed a large right internal iliac artery aneurysm. There was a suspicious finding of a fistula within the colon in the CT, but it was undetected in the preoperative sigmoidoscopy. Furthermore, operative findings showed a protruding retroperitoneal mass adhering to the mesentery of the sigmoid colon. During aneurysm resection, the presence of a fistula was unclear. However, a fistula tract, devoid of any infectious bacteria such as tuberculosis, was found in the specimen after colon resection. After a recovery period of approximately one week, the patient was discharged from the hospital without any unusual findings on the post-operative CT. Sigmoid colonic fistulas arising from iliac artery aneurysms are rare. Also, diagnosis may be delayed in special circumstances wherein a patient routinely uses a bidet.

## 1. Introduction

Primary aneurysmal fistulas of the internal iliac artery involving the lower gastrointestinal tract are rare but have been mentioned in limited case reports. However, this type of fistula differs from a vascular treatment-induced fistula, the so-called secondary aorto-enteric fistula [1]. A secondary aorto-enteric fistula occurs due to routine interventions being commonly performed for a primary aorto-enteric fistula.

Herein, we present a case of the successful treatment of a fistula between an internal iliac artery aneurysm (IIAA) and the sigmoid colon, a cause of anemia that was not accompanied by hematochezia and abdominal pain, which was detected during a routine medical examination.

## 2. Case Report

All procedures performed in this study were in accordance with the ethical standards of the institutional and/or national research committee and with the 1964 Helsinki Declaration and its later amendments or comparable ethical standards. Informed consent was obtained from the patient for the publication of this case report and the accompanying images.

**Figure 1:**
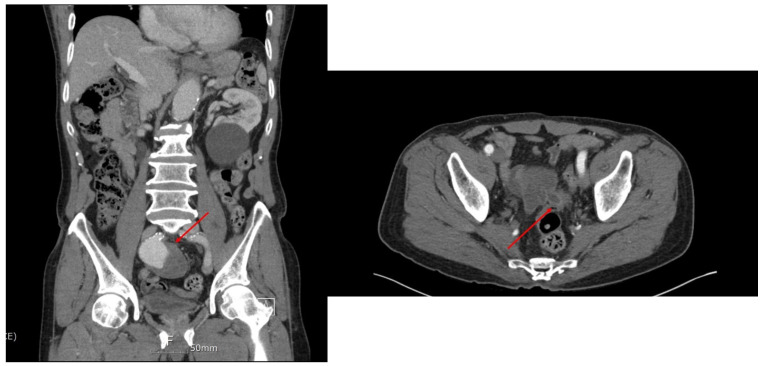
Pre-operative computed tomography showing internal iliac artery aneurysm and fistula tract in sigmoid colon.

A 77-year-old man presented at a local hospital for a general medical examination. He had a history of angina with three vessel diseases. As the initial laboratory tests showed anemia without any symptoms such as dizziness, no transfusion was administered. The conjunctiva was pale, but there was no palpable mass observed during the abdominal examination, and the digital rectal examination was unremarkable for hemorrhage. Abdominopelvic computed tomography (CT) was performed to evaluate the anemia, which revealed a large aneurysm in the right internal iliac artery. The patient was transferred to our tertiary hospital. Upon admission, his vital signs were stable and the hematological tests were normal, except for the hemoglobin level. To determine a precise treatment plan, such as endovascular repair of the aneurysm, aortic CT was performed to confirm the anatomy. The findings were presumed to be aortic colon fistulae that were not observed in the CT (Figure 1). The right internal iliac artery was saccular and 7.5 × 5.1 cm in size. Additionally, the right common iliac artery showed aneurysmal changes of 2.1 cm, which was 1.5-fold larger than those of the left common iliac artery. The right ureter showed dilatation due to the compression of the aneurysm. Despite these findings, the patient did not complain of dysuria or hematochezia. First, endovascular repair of the aneurysm and exclusion of the internal iliac artery with coil embolization were considered; the plan was changed to open repair considering the possibility of colonic fistula infection. Interestingly, sigmoidoscopy did not reveal an opening of the fistula. Our initial intention was to resect the internal iliac artery aneurysm; an additional sigmoidectomy was planned in the event that a fistula tract was discovered.

The surgical procedure was initiated with the patient under general anesthesia. The patient was in a supine position, and an incision was made in the midline, extending from 5 cm above the umbilicus to the pubic bone. Upon repositioning the patient’s small intestine into the upper abdomen, a huge aneurysm was observed.

**Figure 2:**
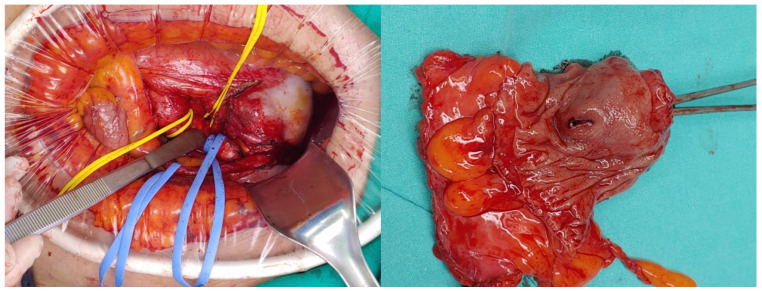
Gross findings and fistula tract with sigmoid colon.

**Figure 3:**
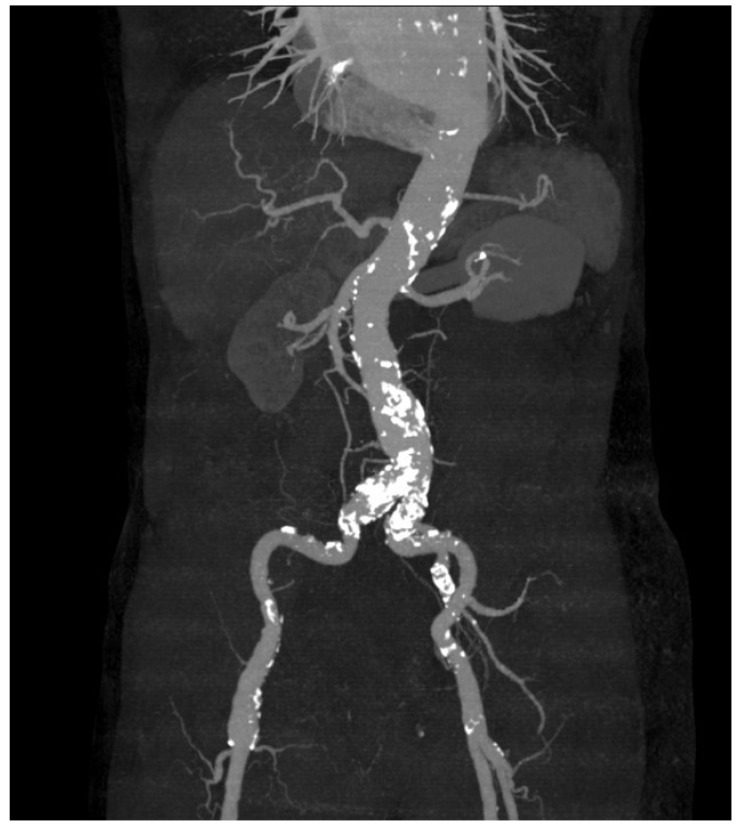
Post-operative computed tomography findings.

Figure 2 shows the operating field. A large IIAA was located anterior to the pelvis. The sigmoid colon was displaced to the left pelvis by the aneurysm. In order to control the bleeding that may occur from the aneurysm, the proximal of the internal iliac artery was first dissected and then ligated. The fistula tract in the sigmoid colon could not be identified until the aneurysm was completely removed from the pelvic cavity. The aneurysm severely adhered to the mesentery of the sigmoid colon, and the fistula tract could only be identified after the mesentery was sufficiently dissected to expose the serosa of the colon. A sigmoid colon measuring approximately 10 cm in length was resected, including the mesentery, which was invaded by an aneurysm. The distal branch of the inferior mesenteric artery was ligated while preserving the left colic artery. Finally, an end-to-end anastomosis between the distal descending colon and proximal rectum was performed, and the operation was terminated. The aneurysms exhibited atherosclerosis with rupture and granulomatous inflammation. The colonic fistula was a false type with chronic inflammation and perforation. Infectious bacteria, such as tuberculosis, were not identified. After a recovery period of approximately one week, the patient was discharged from the hospital without any unusual findings following the post-operative CT (Figure 3). One year later, the patient remained asymptomatic, and treatment was discontinued.

## 3. Discussion

An iliac artery aneurysm is a very rare disease with unknown pathophysiology [2,3]. IIAA rupture within the colon is an equally rare complication, leading to fatal outcomes such as severe rectal bleeding [4,5]. In general, iliac aneurysms are challenging to diagnose based on clinical examinations owing to their deep location within the small pelvis. Additionally, they frequently present without symptoms and may manifest as mass effects that compresses surrounding organs within the pelvis [6]. In terms of clinical manifestation, gastrointestinal bleeding has mainly been reported in previous reports, and it is not clear in the case of abdominal pain or a palpable pulsating mass. In this case, the patient did not complain of any symptoms, and the disease was diagnosed during an incidental evaluation for anemia. Aneurysm-related symptoms include hydronephrosis, lower extremity claudication, and pain or numbness [7,8,9,10]. Following the completion of all treatments, the patient complained of mild right flank pain that might have been due to right ureteric hydronephrosis. In the case of a fistula within the rectum, there is a quiescent period, making it difficult to confirm prior to sudden bleeding, and there are few reports of successfully treated cases [4,5].

Interestingly, the patient’s use of a bidet hampered the diagnosis of hematochezia. The patient was anemic to the extent that a blood transfusion was required. However, a digital rectal examination performed in the emergency department did not reveal evidence of bleeding. It is believed that the aneurysm-related fistula was the cause, given that the anemia did not recur one year after the aneurysm surgery. The cause of chronic anemia may have been intermittent hematochezia [11] due to bowel motility through the fistula, which may have remained undetected in the emergency department. Moreover, the patient may have experienced hematochezia yet failed to recognize the associated symptoms because of their bidet use.

Without screening for anemia, the patient could have unknowingly bled to death. The contrast-enhanced CT revealed that the aneurysm adhered to the colon and the aneurysmal sac was filled with air. Colonoscopy has the advantage of excluding other colonic diseases, including diverticular disease, cancer, and colitis, by identifying rectal ulcers with bleeding. No fistula lesions were found during the sigmoidoscopy, thereby necessitating a gross examination of the surgical field.

Treatments for IIAAs with fistulae are controversial, and there are no optimal methods. Owing to the complexity of the pelvis, the treatment of aneurysms is challenging. Recent developments in the percutaneous technique have reduced the difficulty of extensive pelvic dissection [12,13]. However, in cases of suspected mycotic aneurysm or fistula infections, such treatment has limited use. Previous reports treating aneurysm fistulae in the colon stated that it was important to prevent the contamination of the operative field considering the opening of the bowel [14,15]. In this case, it was planned to completely resect the aneurysm and colon with the fistula to control infections. Infectious sources, including syphilis and tuberculosis, can cause aneurysms [16,17]. Previous studies have reported that the Staphylococcus, Escherichia, and Klebsiella species could be infective causes [18]. In this patient, the cause was presumed to be an aneurysm caused by atherosclerosis and not bacterial infections.

IIAAs with fistulae are rare, and they are difficult to diagnose and treat because of their high mortality rate. An accurate diagnosis and systematic treatment plan should be established to obtain good outcomes. The unusual circumstances of using a bidet may further delay the diagnosis of a sigmoid colon fistula from an IIAA.

## Data Availability

The data sets used in the present study are available from the corresponding author upon reasonable request.
